# A Non-Lévy Random Walk in Chacma Baboons: What Does It Mean?

**DOI:** 10.1371/journal.pone.0016131

**Published:** 2011-01-13

**Authors:** Cédric Sueur

**Affiliations:** 1 Department of Ecology and Evolutionary Biology, Princeton University, Princeton, New Jersey, United States of America; 2 Unit of Social Ecology, Free University of Brussels, Brussels, Belgium; Cajal Institute, Spain

## Abstract

The Lévy walk is found from amoebas to humans and has been described as the optimal strategy for food research. Recent results, however, have generated controversy about this conclusion since animals also display alternatives to the Lévy walk such as the Brownian walk or mental maps and because movement patterns found in some species only seem to depend on food patches distribution. Here I show that movement patterns of chacma baboons do not follow a Lévy walk but a Brownian process. Moreover this Brownian walk is not the main process responsible for movement patterns of baboons. Findings about their speed and trajectories show that baboons use metal maps and memory to find resources. Thus the Brownian process found in this species appears to be more dependent on the environment or might be an alternative when known food patches are depleted and when animals have to find new resources.

## Introduction

Particles suspended in a fluid, air or water move in a random way called Brownian motion [1]–[3]. This rule is also used, however, to explain phenomena in geology, ecology or social sciences. Several studies suggested that animals used random walks as a strategy to find food or reproductive partners by increasing the probability of encountering the respective item [4]–[7].

All random walks are composed of three basic measurements: the waiting time to an area A, the step length between areas A and B and the turning angle. Whereas Brownian random walks are characterised by constant length of steps and waiting times, Lévy walks describe movement patterns characterised by many small steps connected by rare long steps. In the first case, the probability distribution of step length is exponential whereas in the second case it is power-law. This Lévy walk was defined as an optimal strategy for a forager searching without information about its heterogeneous environment with low density food patches [3], [4], [8]. There has been growing interest in the Lévy walk and this strategy is reported in many species such as soil amoebas [9], zooplankton [5], jackals [10], albatrosses [11] and elephants [12]. This similarity between these phylogenetically distant species suggests that random walks are efficient and adaptive. Some studies cast doubt on Lévy walks as an optimal strategy existing in animals, however, first because of methodological shortcomings in the estimation of power-law exponents but also because of the impact of resource distribution and the probability of species' cognitive abilities being sufficient to find this strategy [3], [13]–[15].

Primates are known to use high cognitive processes in their foraging and travel decisions [16]. The use of spatial memory to remember patterns of resource availability and distribution was shown in several species (red-tailed monkeys, *Cercopithecus ascanius* [17], Japanese macaques, *Macaca fuscata* [18], long-tailed macaques, *M. fascicularis* [19], white-faced capuchins, *Cebus capucinus* [20], brown capuchins, *C. apella nigritus* [21]). Thus, it is expected that primates would not walk “randomly” in their environment. Studies about random walks in primates are scarce but recent studies showed that spider monkeys (*Ateles* geoffroy [14], [22]) and hamadryas baboons (*Papio hamadryas hamadryas* [23]) used Lévy walks in their foraging movements. Probability distributions of their step lengths as well as of their waiting times follow a power law. Other studies also showed that baboons used the shortest linear route to travel from one location to another and that they speeded up as they approached a water or food source, indicating goal-directed and mental map processes [24]–[27]. Consequently, the definition provided by Viswanathan and colleagues [28] of the Lévy walk being an optimal search strategy without prior information is questionable.

Here, I study the movements of a group of chacma baboons (*P. ursinus*) in their natural environment. I will assess whether distributions of step lengths and also times correspond to the Brownian or Lévy walk. I will also study trajectories and speed of animals in order to assess whether they use more cognitive processes.

## Methods

### Study site and subjects

Data on chacma baboons were scored at the Wildcliff Nature Reserve, Western Cape, South Africa (33.959997°N, 21.034478°E) from May to August 2009. The reserve is a mountain wilderness reserve consisting of deep ravines with afro-mountain forest, rocky mountain tops and high meadows of fynbos. An invasive plant, the black wattle, and a grassy meadow are also present. Three groups of chacma baboons populate this reserve and its surroundings. At the time of the study, the study group consisted of between 90 and 100 individuals (about 9.1% were males, 37% were females without babies (<1year), 5.6% were females with babies, 16.5% were sub-adults (4-6 years old) and 31.8% were juveniles (1–3 years old)). During this study, animals were just observed. No animal handling or invasive experiment was done on studied subjects. We declare that our study is in full accordance with the ethical guidelines of our institution with the approval of the latter (certificate number: 67-339, French Republic, Bas-Rhin County Hall, French veterinary services). Our experiments comply with European animal welfare legislation.

### Data collection

During the study period I, accompanied by a field assistant, scored the location of the baboons' group from dusk (about 7:00) to dawn (about 17:00). The location of the baboon group was defined as the geographical centre of the band [23], [26] by means of a GPS Sirf 3 Holux. I scored locations every ten minutes. The GPS accuracy is inferior to two meters. Activity of baboons, the number of individuals observed in each activity and the kind of vegetation were also scored by means of Cyber Tracker 3.0 (Cyber Tracker Conservation, Bellville, SA) with a PDA Asus 620. The activities of baboons included moving (locomotion including walking, running, climbing and jumping), foraging (reaching for, picking, manipulating, masticating, or placing food in mouth, as well as manipulating the contents of a cheek pouch), resting (body stationary, usually sitting or lying down) and socialising (playing, grooming, sexual and aggressive behaviour) [29], [30]. I defined five kind of vegetations by characterising the dominant species; fynbos, black wattle, grass, pine, afro-mountain forest. This vegetation study was done prior to the baboon study. The sleeping sites were identified by me, my field assistant and Paula Pebsworth (personal communication). The clay site was identified by Paula Pebsworth (personal communication). I only kept data for which all samples were obtained throughout the day. I obtained eighteen days' worth of observations and 740 samples.

### Data analysis

A step was defined as an interval in which any or both of the coordinates in two consecutive samples differed. The length *L* of a step was calculated with the formula:
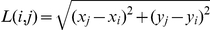

*x* and *y* are respectively the latitude and longitude of points *i* and *j*.

The length expressed in decimal degree was then transformed in meters.

I carried out survival analysis to study the inverse cumulative distribution of step lengths. This distribution of step lengths was compared with exponential, power and Weibull law [31].

I also calculated the mean squared displacement using the following procedure: for each day the length of a line joining the first recorded location (commonly the sleeping site) of the group with its location at different steps (every ten minutes). Then, all-day squared displacements were averaged at intervals of 30 minutes, from 6:30 to 18:00. I also calculated the mean path length every 30 minutes. Waiting times were calculated from the number of samples in which the group did not change position. I carried out survival analysis to study the inverse cumulative distribution of waiting times. This distribution was compared with exponential and power law. All these distributions were analysed with curve estimation tests. The results for Weibull distribution were only indicated when the correlation coefficient between the Weibull distribution and the tested variable was higher than the one with exponential distribution. I also calculated the mean path length for each kind of vegetation and correlated it with the foraging time animals spent in each kind of vegetation. Differences of mean path length between kinds of vegetation were analysed with a median test (detection of differences in shape and location). Only samples where animals changed the type of vegetation were kept for this analysis. Correlation between path length and foraging time was tested with a curve estimation test. All tests were done with SPSS 10.0., α = 0.05.

## Results

**Figure 1:**
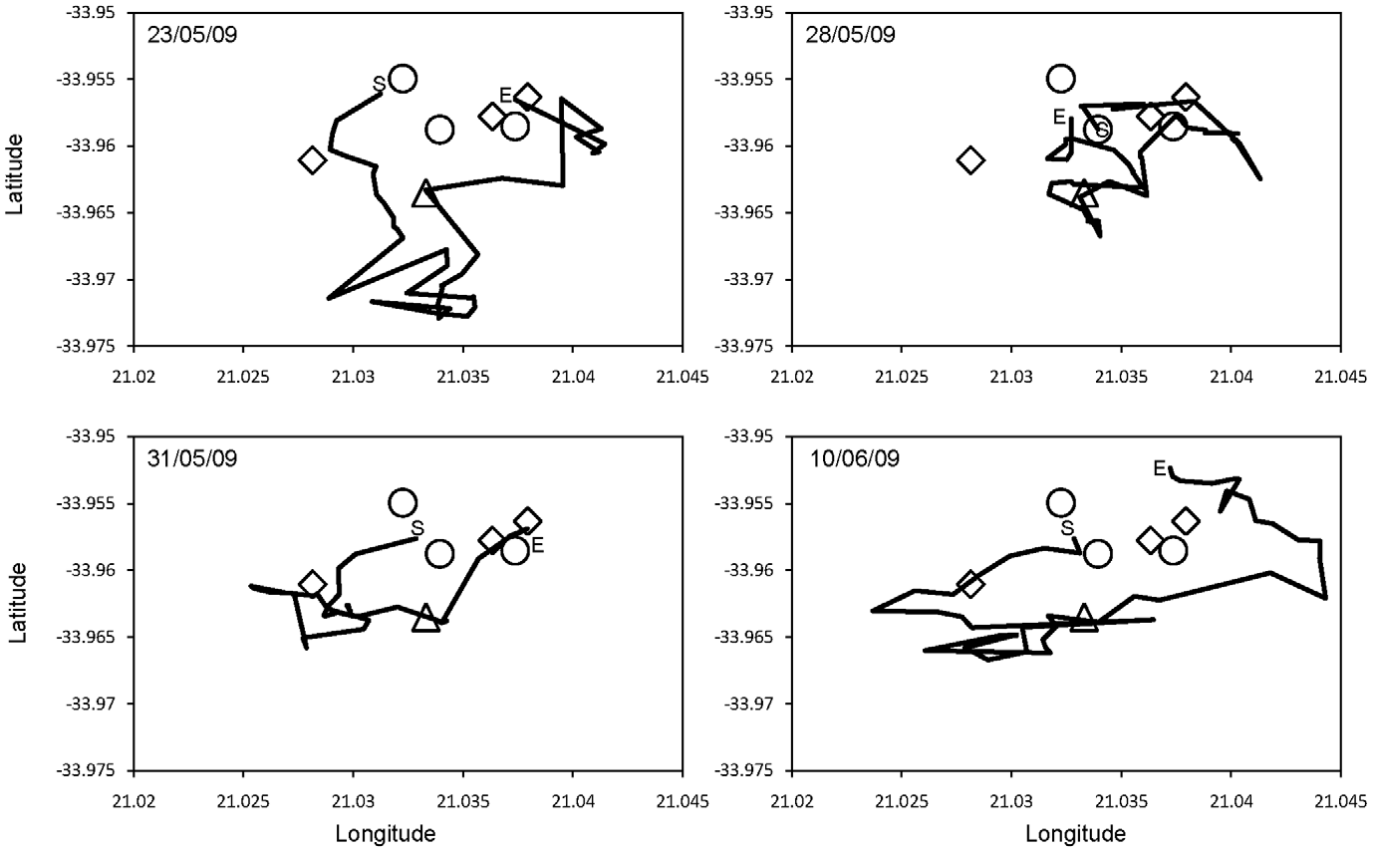
Four daily trajectories of the study group of chacma baboons. S represents the location where animals started their daily travel. E represents the location where animals ended their travel. Circles represent sleeping sites. Squares are waterholes. The triangle is a clay site.

Figure 1 shows four daily trajectories of the group. These trajectories seem to be made up of steps of different lengths. Moreover, animals seemed to go first to a waterhole (observed in 100% of cases), and then to go everyday to a clay site (observed in 83.3% of cases), to forage and eventually to come back at the end of the day to their sleeping site, doing a kind of ellipse all along their home range.

**Figure 2:**
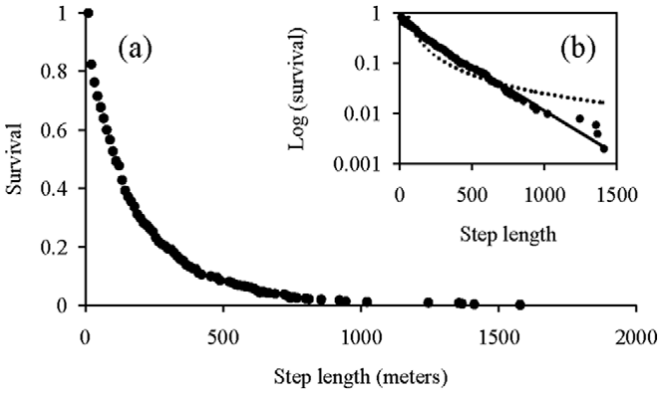
Inverse cumulative distribution of step lengths. The inset (b) shows the log plot of the same data. Circles are observed data. The continuous line is the theoretical exponential curve representing the Brownian random walk. The dotted line is the theoretical power curve representing the Lévy walk.

The distribution of step lengths for all observations fitted better with exponential law (R^2^  = 0.98, F_1,69_  = 4060, p<0.00001, *y = 0.6384e^−0.004x^*, Figure 2) than to power law (R^2^ = 0.81, F_1,69_  = 291, p<0.00001, *y = 150.86x^−1.256^*, Figure 2). More specifically, this distribution better fits with a Weibull function (R^2^  = 0.99, F_1,69_  = 12481, p<0.00001, 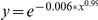). The Weibull distribution is a continuous probability distribution with a tail heavier than the one of exponential function [31]. Similarly, analysis of step lengths between 10:30 and 13:30 shows that the distribution more followed an exponential law (R^2^ = 0.97, F_1,234_  = 8867, p<0.00001, *y = 1.4419e^−0.001x^*) that a power one (R^2^ = 0.61, F_1,234_  = 360, p<0.00001, *y = 33.501x^−0.639^*). This confirms the high variability of step lengths. The walk, however, seems to be more Brownian than a Lévy one because of the exponential distribution.

**Figure 3:**
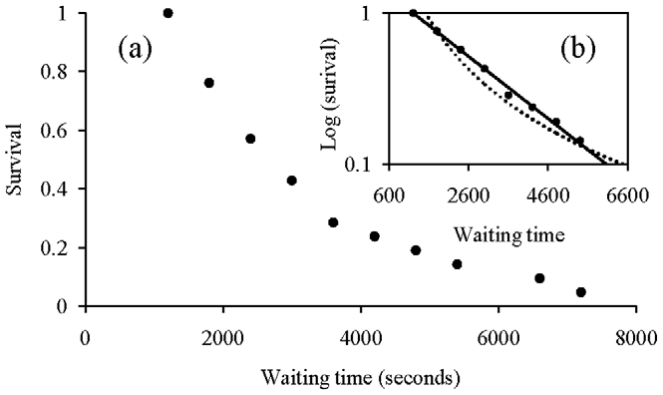
Inverse cumulative distribution of waiting times. The inset (b) shows the log plot of the same data. Circles are observed data. The continuous line is the theoretical exponential curve representing the Brownian random walk. The dotted line is the theoretical power curve representing the Lévy walk.

The distribution of waiting times also shows high variability. Chacma baboons were stationary for a minimum of about ten minutes to a maximum of about two hours (Figure 3). The distribution of these waiting times seems however to follow an exponential curve (R^2^ = 0.99, F_1,8_ = 1597, p<0.00001, *y = 1.7668e^−0.0005x^*, Figure 3) rather than a power one (R^2^ = 0.87, F_1,8_ = 57, p<0.00001, *y = 113303x^−1.589^*, Figure 3). Here again, animals seem to walk according to a Brownian process rather than a Lévyesque one because of the exponential distribution.

**Figure 4:**
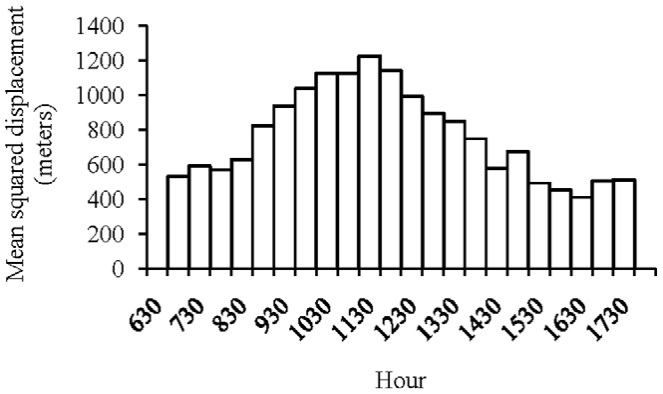
Mean squared displacement of the group at different times of the day.

The squared displacement of the group shows that animals go away from one of their sleeping sites in the morning but come back to it for the evening (Figure 4). This parabolic shape is confirmed by a curve estimation test (R^2^ = 0.65, F_1,22_ = 38, p<0.00001, *y = −0.003x^2^ +7.186x−3311.3*). The maximum squared displacement is at 11:30.

**Figure 5:**
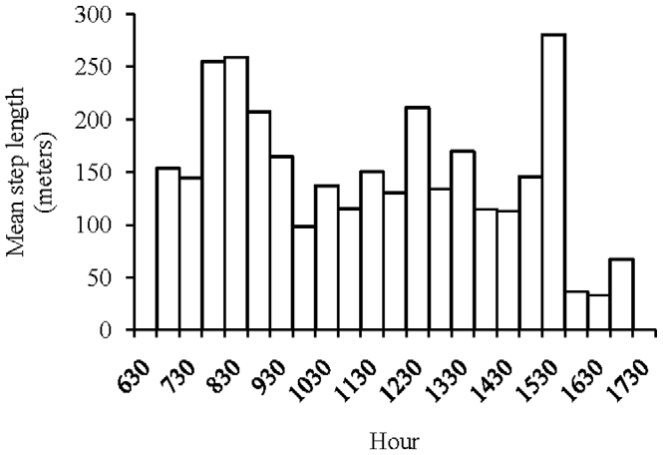
Mean step length of the group at different times of the day.

The distribution of the mean step length per hour showed that animals speeded up when leaving their sleeping site in the morning (maximum at 8:30 a.m.) and when coming back to it at the end of the afternoon (maximum at 15:30 p.m.) (Figure 5). Indeed, the curve from 8:30 a.m. to 15:30 p.m. follows a parabolic law (R^2^ = 0.36, F_1,14_ = 12, p = 0.004, *y = 0.0008x^2^−1.886x +1258.5*).

Finally, the mean step length differs according to the kind of vegetation animals are going to (N = 490, χ^2^ = 13.7, p = 0.032). Moreover, this mean step length is correlated with the time animals foraged in each kind of vegetation (R^2^ = 0.89, F_1,4_ = 32, p = 0.005, *y = 0.00003e^0.0493x^*). This shows that the more animals need to eat a specific species (correlated with the foraging time in each type of vegetation), the more they speed up to go to the respective area.

## Discussion

This study shows that chacma baboons present evidence of random walks in their daily trajectories. Distributions of step lengths as well as waiting times follow exponential laws, suggesting a Brownian process. These baboons, however, also seem to have a routine: starting every day from their sleeping site, going first to a waterhole, going in almost each case to a clay site and then coming back to their sleeping site for the evening. This result suggests that animals do not travel randomly in their environment but have a mental map. These two apparent opposite results should be discussed in order to understand what really happens in baboons' minds.

Viswanathan and colleagues [4], [6], [28] suggest that the Lévy walk is an optimal food research strategy for animals and therefore should be found in almost all species. This study shows, however, that this group of chacma baboons does not use the Lévy walk but rather a Brownian process. Actually, instead of being an optimal strategy, the Lévy walk may also be a pattern emerging from the environment. Distribution of food patches or prey might lead animals to change from one kind of random walk to another one, from Brownian to Lévy walk for instance. Humphries et al. [3] found that movement patterns in marine predators depend on environmental context. The Lévy walk should be adopted when resources are sparse and unpredictably distributed whereas Brownian movement is efficient when resources are abundant and homogeneously distributed. Consequently, Boyer et al. [15] explained how the Lévy walk found in spider monkeys is reproduced by a simple model where animals forage in a spatially disordered environment with patches of heterogeneous size distribution. The previous findings and the results of this study suggest that random walks by chacma baboons depend more on resource distribution in their environment than on a cognitive process. This should also explain why the Lévy walk and not the Brownian one was found in hamadryas baboons: patches in the environment of the studied hamadryas baboons were sparser than those in that of chacma baboons [23].

Another striking point is finding random walks in species known for their high cognitive abilities [32]. Similar questions were also raised in studies on spider monkeys [14] and hamadryas baboons [23]. Some findings in spider monkeys do not point to a purely Lévy walk in this species but suggest that animals also use mental maps. Spider monkeys come back almost every night to specific sleeping sites. The shape of their home range looks like a circle. They often use the same route when returning to the sleeping site. These results suggest a knowledge base in their memory that animals use. This is contradictory with Lévy walk as an optimal research strategy. This assumption was confirmed by a model where animals using memory and a metal map to search for food in a spatially disordered environment exhibited random walks [15]. The studied chacma baboons show similar cognitive processes. Their daily trajectory looks like an ellipse going through specific locations such as waterholes and clay sites and with sleeping sites as start and end points. Analyses of their step lengths also show increasing speed when baboons leave or arrive at their sleeping site. Baboons also speed up when going to important food locations. This result suggests goal directness in animals: they know where to go. Noser & Byrne [27] found similar results in another group of chacma baboons. By studying travel speed and route linearity of baboons, they found that animals seem to plan their journeys and actively choose their out-of-sight resources, reaching them in an efficient and goal-directed way. These characteristics allow us reasonably to infer the presence of mental maps and use of memory [26], [33], [34]. Chacma baboons in this study should use the same cognitive processes to find their way but it is also possible that sometimes animals use a random walk to find new and unknown sources, switching between the two processes [14].

This study confirms that random walks, whether Brownian or Lévyesque, should not be considered as the only food strategies animals have. Existence of these processes in animals may depend on food patch distribution but may also be used as an alternative strategy to find new resources when known food patches are depleted. Instead of only considering resources, we also need to assess how predation risk influence movement patterns of animals.
